# Targeted therapy of human leukemia xenografts in immunodeficient zebrafish

**DOI:** 10.1038/s41598-021-85141-5

**Published:** 2021-03-11

**Authors:** Ranganatha R. Somasagara, Xiaoyan Huang, Chunyu Xu, Jamil Haider, Jonathan S. Serody, Paul M. Armistead, TinChung Leung

**Affiliations:** 1 The Julius L. Chambers Biomedical/Biotechnology Research Institute, North Carolina Central University, North Carolina Research Campus, Kannapolis, NC 28081 USA; 2 Department of Biological & Biomedical Sciences, North Carolina Central University, Durham, NC 27707 USA; 3 Division of Hematology/Oncology, Department of Medicine, Lineberger Comprehensive Cancer Center, University of North Carolina at Chapel Hill, Chapel Hill, NC 27599 USA

**Keywords:** Cancer models, Cancer therapy, Haematological cancer

## Abstract

Personalized medicine holds tremendous promise for improving safety and efficacy of drug therapies by optimizing treatment regimens. Rapidly developed patient-derived xenografts (pdx) could be a helpful tool for analyzing the effect of drugs against an individual’s tumor by growing the tumor in an immunodeficient animal. Severe combined immunodeficiency (SCID) mice enable efficient in vivo expansion of vital tumor cells and generation of personalized xenografts. However, they are not amenable to large-scale rapid screening, which is critical in identifying new compounds from large compound libraries. The development of a zebrafish model suitable for pdx could facilitate large-scale screening of drugs targeted against specific malignancies. Here, we describe a novel strategy for establishing a zebrafish model for drug testing in leukemia xenografts. We used chronic myelogenous leukemia and acute myeloid leukemia for xenotransplantation into SCID zebrafish to evaluate drug screening protocols. We showed the in vivo efficacy of the ABL inhibitor imatinib, MEK inhibitor U0126, cytarabine, azacitidine and arsenic trioxide. We performed corresponding in vitro studies, demonstrating that combination of MEK- and FLT3-inhibitors exhibit an enhanced effect in vitro. We further evaluated the feasibility of zebrafish for transplantation of primary human hematopoietic cells that can survive at 15 day-post-fertilization. Our results provide critical insights to guide development of high-throughput platforms for evaluating leukemia.

## Introduction

SCID mice have a homozygous mutation at the *Prkdc* gene, required for DNA repair and for sealing the double-stranded DNA breaks. In the absence of functioning *Prkdc*, T cell receptor and immunoglobulin genes cannot rearrange, resulting in mice with severe T and B cell immune deficiency. Similar to mice, deficiency of *prkdc* in zebrafish results in loss of mature T and B cells^1^. *Prkdc* mutant fish are viable as homozygotes and survive injury after cell transplantation. This mutant efficiently engrafts fluorescently labeled normal and malignant cells, such as T-cell acute lymphoblastic leukemia (T-ALL)^1^.

A high-throughput experimental animal leukemia model with easy access for live imaging, quick turn-around-time for experimental procedures, and easy manipulation for large-scale drug testing would be a major breakthrough, both for understanding leukemogenesis and developing effective personalized treatments from drug testing. These characteristics are particular strengths of the zebrafish model. Zebrafish with *casper* mutant background are optically transparent, enabling real-time observation of transplanted cancer cells in live embryos^2^. The zebrafish genome is fully sequenced and gene analysis techniques are well established, with resources available through the Zebrafish Model Organism Database (ZFIN; https://zfin.org/) and the Wellcome Trust Sanger Institute (https://www.sanger.ac.uk/science/data/zebrafish-genome-project). Zebrafish genes and proteins are 70–80% identical to humans. In zebrafish, as in other vertebrates and humans, blood cells are formed from the mesoderm near the aorta, later at the posterior blood island (primitive hematopoiesis) and the caudal hematopoietic tissues (CHT) (definitive hematopoiesis), which forms a transient site of hematopoiesis; blood cells finally migrate to the thymus and pronephros (kidney marrow)^3–5^. The development of the hematopoietic stem/progenitor cells (HSPC) is readily accessible by genetic manipulation or chemical modulation in zebrafish^6–8^. The conserved mechanism^9,10^ of HSPC development makes it possible to develop a zebrafish model that supports human hematopoietic and leukemia cells in vivo. The development of such advanced techniques as transgenesis and knock-in approaches over the last few decades has made zebrafish a compelling model for drug testing.

Zebrafish/tumor xenografts have been established by other researchers and our laboratory to visualize and functionally analyze the role of cancer cells and monitor their interactions with the microenvironment during tumor angiogenesis, metastasis and in response to drug treatment^11–18^. Zebrafish embryos lack adaptive immunity in the early stages of development; therefore, cell-mediated host versus graft rejection can be circumvented, simplifying engraftment—even with primary patient-derived samples—without the need for immunosuppression^19,20^. The optical transparency of the zebrafish simplifies xenotransplantation of solid tumors; moreover, it enables imaging of cancers with single-cell resolution and facilitates engraftment study in the immunocompromised zebrafish model (*prkdc* and *rag2* mutant lines)^1,21,22^. Human-derived hematopoietic progenitors can be transplanted into zebrafish to visualize human HSPC trafficking in vivo^23–25^. Zebrafish can also be used for functional analysis, genetic manipulation, and live imaging of leukemia cells as an alternative model for studying leukemia and other hematopoietic proliferative disorders^21,23,25–27^. Many molecular and cellular components that operate during tumorigenesis are conserved between zebrafish and mammals. Further, a wide range of pharmacologically active compounds elicit physiological responses in zebrafish embryos comparable to those in mammalian systems, making zebrafish ideal for identifying clinically relevant genes and compounds that regulate tumor progression^28^. In addition, zebrafish models require much less experimental time compared to mouse models, thereby shortening turn-around-times for drug testing. This, combined with the capability of optical imaging, makes zerbrafish highly attractive models for investigating different drug combination therapies against patient-derived xenografts for leukemia^2,29–33^. Furthermore, the zebrafish xenograft model can be a useful tool for translational research: for example, drug screening on patient-derived xenografts in preclinical settings^34,35^.

## Results

### Xenotransplantation of leukemia cells into SCID zebrafish for drug study

**Figure 1:**
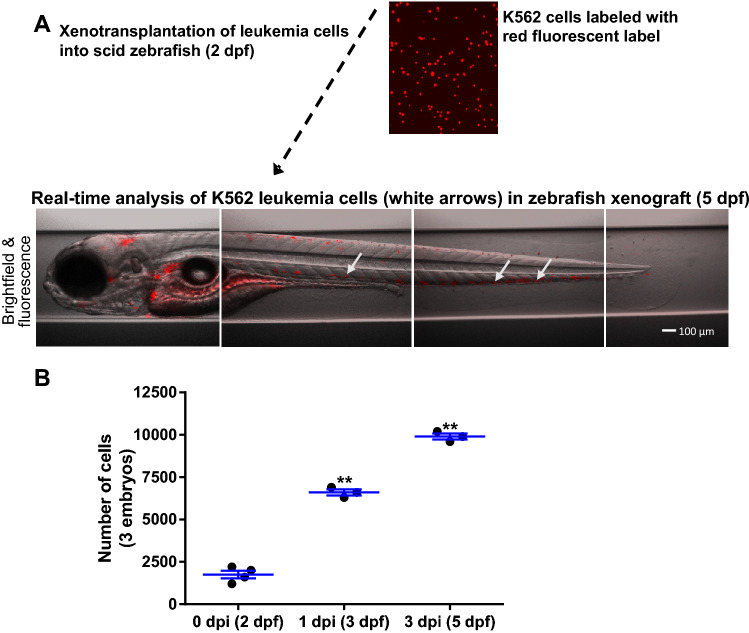
Xenotransplantation of leukemia cells into SCID zebrafish. **(A)** Microscopic observation of K562 cells at 5 dpf zebrafish xenograft. (**B)** The 5 dpf zebrafish xenograft embryos were dissociated and labeled leukemia cells were analyzed manually using a hemocytometer. Error bars were calculated based on biological replicates in the experiment. Quantitative values were means ± SEM from 3–4 independent groups of 10 individuals at 3 dpf and 5 dpf. Control at 2 dpf represented the initial transplanted cells into the zebrafish from two separate experiments and showed a consistent amount of transplanted cells. Significance *p* values were determined as *p ≤ 0.05, **p ≤ 0.01 and ***p ≤ 0.001.

To develop zebrafish as a novel xenograft model for prioritizing cancer therapeutics, we transplanted fluorescently labeled chronic myelogenous leukemia (CML) K562 cells into 2 dpf (day-post-fertilization) SCID zebrafish embryos. As a control, zebrafish xenografts at 2 dpf were dissociated, analyzed, and cell numbers evaluated to obtain a baseline number of human cells just after transplantation. These 2 dpf xenografts were used as the 0 dpi (day-post-injection) control group, which represented the initial amounts of human cells being transplanted by this microinjection protocol and were used to compare at later time points after cell proliferation inside the zebrafish host. All groups of xenografts were incubated at 33 °C until 3 dpf and 5 dpf. The xenograft embryos were dissociated on the respective days and xenografted human cells were analyzed. Our results showed that the transplanted human K562 cells were significantly increased both at 3 dpf and 5 dpf (p ≤ 0.01) as compared to the cell numbers at 2 dpf (Fig. 1A,B). Figure 1A shows a representative image of the fluorescently labeled K562 cells in the zebrafish xenografts at 5 dpf, and the human cancer cells can be quantified inside the zebrafish embryos (Fig. 1B).

### Both malignant and normal human hematopoietic stem/progenitor cells proliferate in zebrafish xenograft embryos

**Figure 2:**
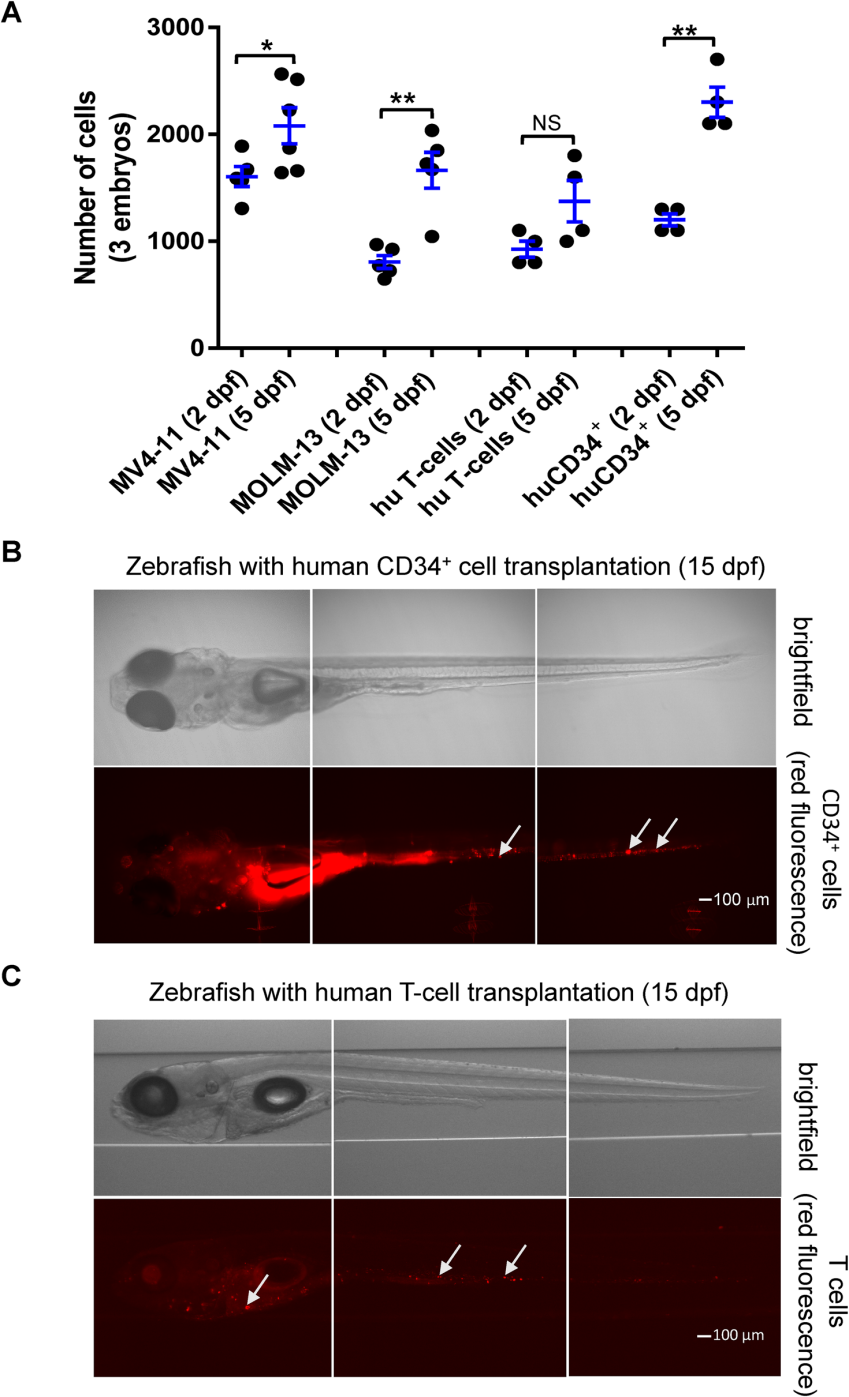
Xenotransplantation of AML cells and human primary cells into SCID zebrafish. **(A)** Proliferation of MV4-11, MOLM-13, human CD34^+^, and CD3^+^ T-cells in zebrafish xenografts started from 2 dpf. The zebrafish xenograft embryos were dissociated at 2 dpf and 5 dpf, and labeled leukemia or human primary cells were analyzed by ImageXpress Pico. (**B)** Representative images of zebrafish xenografts with CD34^+^ HSPC cells at 15 dpf. (**C)** Representative images of zebrafish xenografts with CD3^+^ T-cells at 15 dpf. Quantitative values were means ± SEM. Sample size was between 4 and 6 samples per experimental group and each sample contained 3 xenografts. Significance *p* values were determined as *p ≤ 0.05, **p ≤ 0.01 and ***p ≤ 0.001.

Next, we evaluated the ability of both malignant and non-malignant hematopoietic cells to engraft in zebrafish embryos. We transplanted human malignant leukemia cell lines, human CD34^+^ HSPC and human T cells, into zebrafish embryos. Both malignant acute myeloid leukemia (AML) cell lines (MV4-11 and MOLM-13) and hematopoietic cells from normal controls (human CD34^+^ HSPC) were increased in cell numbers at 5 dpf after transplantation inside the zebrafish xenografts (Fig. 2A), while engrafted human T cells also survived within the zebrafish host but cell proliferation was not statistical significance (Fig. 2A). Thus, zebrafish can be used to engraft human AML cell lines, HSPC, and T cells from normal donors. Our results also demonstrate that the engrafted human HSPC and T-cells can persist for at least 15 dpf (Fig. 2B,C).

### Proliferation of K562 cells in SCID zebrafish xenografts is inhibited by drug treatment

**Figure 3:**
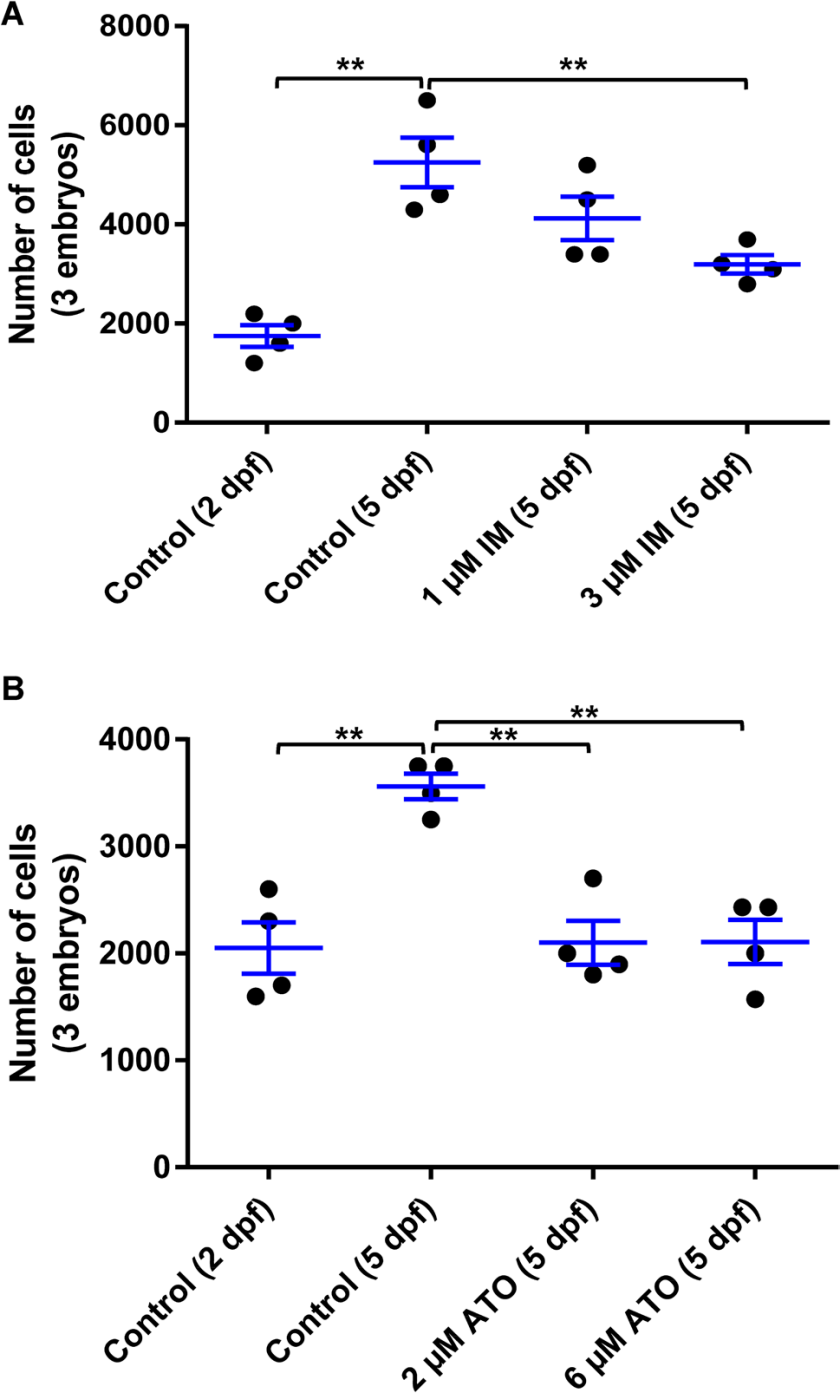
Leukemia xenografts in zebrafish and drug treatment. Proliferation of K562 cells in the zebrafish xenograft model in different groups of IM and ATO treatments started from 2 dpf. The zebrafish xenograft embryos were dissociated (control group at 2 dpf and 5 dpf, treatment group at 5 dpf), and labeled leukemia cells were analyzed manually using a hemocytometer. (**A)** The total K562 cells in zebrafish xenografts after IM treatment (0, 1, and 3 µM). (**B)** The total K562 cells in zebrafish xenografts after ATO treatment (0, 2, and 6 µM). Quantitative values were means ± SEM from 4 independent groups of 10 xenografts in each group. Significance *p* values were determined as *p ≤ 0.05, **p ≤ 0.01 and ***p ≤ 0.001.

The ability to evaluate anti-tumor response in zebrafish represents a promising, inexpensive, rapid screening platform for drug treatments. To assess the feasibility of this approach, clinically approved drugs against K562 cells, an erythroleukemia cell line with the BCR-ABL mutation, were evaluated. We began by evaluating the well-established BCR-ABL targeted drug imatinib (IM) on K562 cells. Our results revealed that K562 cell proliferation was suppressed with increasing dosage of IM in zebrafish/K562 xenografts (Fig. 3A). We also evaluated a second anti-cancer drug, arsenic trioxide (ATO): this drug exerted a cytotoxic effect similar to IM on K562 cells in zebrafish xenografts (Fig. 3B). The results showed that the growth of K562 cells were significantly inhibited by both drug treatments as compared to the control group (p ≤ 0.01). ATO is an effective anti-cancer treatment, approved by US Food and Drug Administration (FDA) for treatment of acute promyelocytic leukemia; however, it can be very toxic^36,37^. Our results showed that zebrafish tolerated the drug, indicating that the assay could reveal the effects of anti-cancer activity without obvious toxicity to zebrafish embryos (for example, microscopic observation showed no arrhythmia, normal cardiac heart beat, normal differentiation of neural and blood tissues and no developmental delay; data not shown), which makes the zebrafish platform applicable for potential drug screens of a large chemical library against leukemia.

### Blocking the MEK signaling pathway inhibits the proliferation of MOLM-13 AML cells in zebrafish xenografts

**Figure 4:**
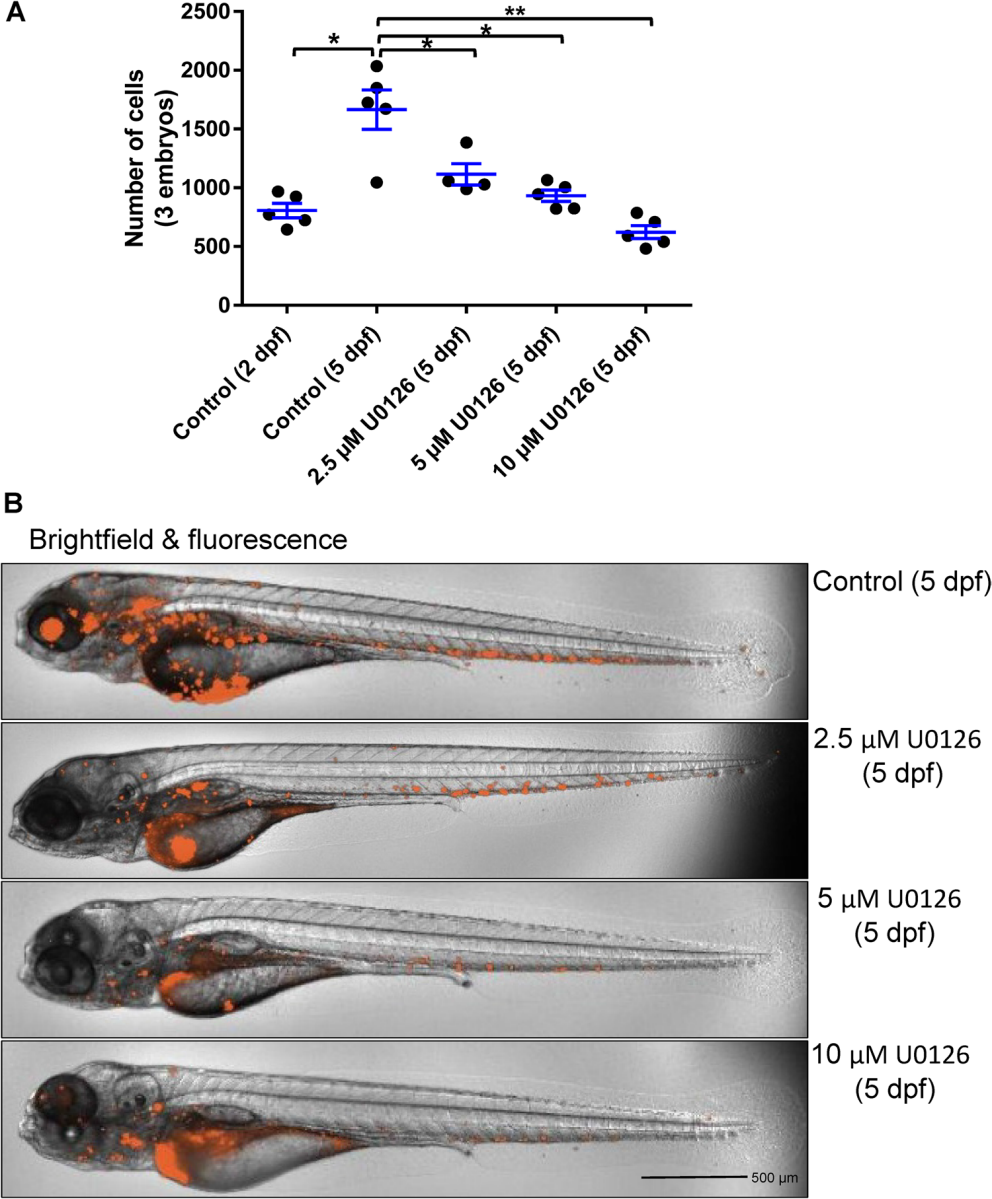
MOLM-13 cells in zebrafish xenografts and U0126 treatment. Proliferation of MOLM-13 cells in the zebrafish xenograft model with U0126 treatment started from 2 dpf. The zebrafish xenograft embryos were dissociated (control group at 2 dpf and 5 dpf and treatment group at 5 dpf), and labeled leukemia cells were analyzed by ImageXpress Pico. (**A)** Total MOLM-13 cells in zebrafish xenografts after U0126 treatment (0, 2.5, 5, and 10 µM). (**B)** Representative images of zebrafish xenografts at 5 dpf with U0126 treatments. Quantitative values were means ± SEM from 4 to 5 independent groups of 3 xenografts in each group. Significance *p* values were determined as *p ≤ 0.05, **p ≤ 0.01 and ***p ≤ 0.001.

**Figure 5:**
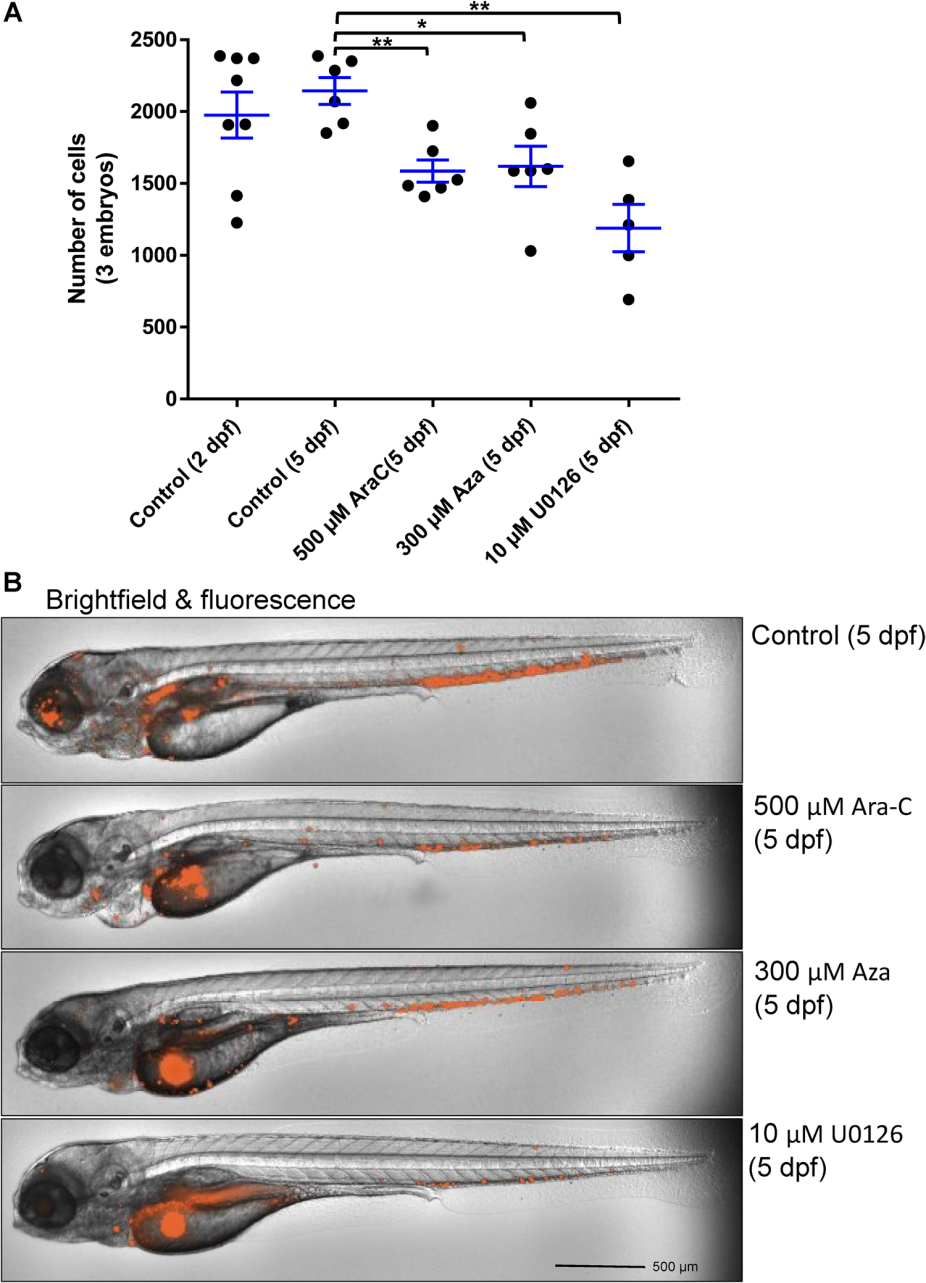
MV4-11 cells in zebrafish xenografts and drug treatment. Proliferation of MV4-11 cells in the zebrafish xenograft model with Ara-C and Aza treatments started from 2 dpf. The zebrafish xenograft embryos were dissociated (control group at 2 dpf and 5 dpf and treatment group at 5 dpf), and labeled leukemia cells were analyzed by ImageXpress Pico. (**A)** Total MV4-11 cells in zebrafish xenografts after Ara-C (500 µM), Aza (300 µM), and U0126 (10 µM) treatment. (**B)** Representative images of zebrafish xenografts with respective concentrations of drug treatment at 5 dpf. Quantitative values were means ± SEM from 5 to 8 independent groups of 3 xenografts in each group. Significance *p* values were determined as *p ≤ 0.05, **p ≤ 0.01 and ***p ≤ 0.001.

To further evaluate the feasibility of using the zebrafish model for screening additional drug targets, we tested whether the MAPK pathway inhibitor and anti-leukemic agents were effective. To validate the zebrafish xenograft model, we evaluated an MEK inhibitor (U0126) as drug compound for MOLM-13 leukemia cells in zebrafish xenografts up to 5 dpf. Our results revealed that U0126 treatment inhibited proliferation of MOLM-13 cells in zebrafish xenografts in a dose-dependent manner as compared with the control group (Fig. 4A). We also imaged the zebrafish xenografts using ImageXpress Pico for U0126 treated xenografts, as shown in representative image in Fig. 4B. The images showed a qualitative result similar to the quantitative cell count analysis after xenograft embryo dissociation. In addition, we evaluated the effect of clinically used anti-leukemic drugs (500 µM Ara-C and 300 µM Aza) against MV4-11 cells along with U0126. These agents also showed similar anti-leukemic activity as compared to U0126 in the zebrafish leukemia xenografts (Fig. 5A,B).

### MEK inhibitor, alone and in combination with FLT3 inhibitor, reduces MV4-11 cell viability in vitro

**Figure 6:**
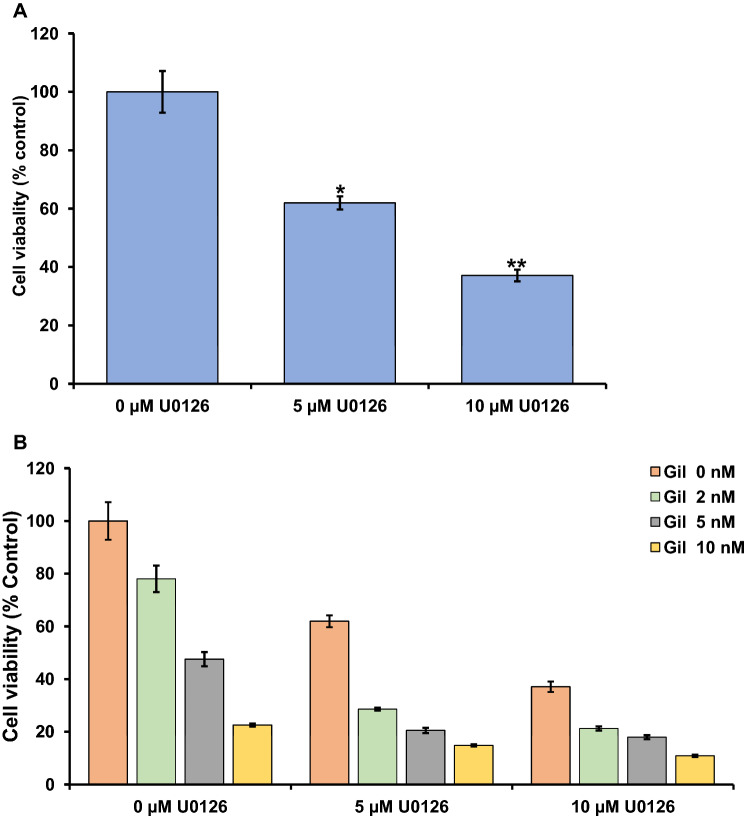
Assessment of U0126 and Gilteritinib induced cytotoxicity in AML cells. MV4-11 cells were treated with different concentrations of U0126 (0, 5 and 10 µM) and Gilteritinib (0, 2, 5 and 10 nM); cells were harvested after 72 h of treatment and subjected to live and dead cell count using ImageXpress Pico. (**A)** Determination of cell viability after U0126 treatment on MV4-11 cells. (**B)** Determination of cell viability after combined treatment of MV4-11 cells with U0126 and Gilteritinib. In each panel, error bars (SEM) were calculated based on biological replicates in the experiment. In all panels, significance *p* values were determined as *p ≤ 0.05, **p ≤ 0.01 and ***p ≤ 0.001.

The mitogen-activated protein kinase (MAPK) pathway is a key integration point in the signal transduction cascade that links diverse extracellular stimuli to proliferation, differentiation, and survival^38^. Inappropriate MAPK activation may also play a role in the leukemic transformation of myeloid cells. Some reports suggest that disruption of the MAPK/ERK pathway (also known as the Ras-Raf-MEK-ERK pathway) has profound functional consequences in AML cell lines and primary AML samples^39,40^. This is consistent with previous study reported that the activation of RAS/MAPK pathway through AXL activation induced resistance to FLT3 inhibitor in AML cells^41^. Hence, inhibition of these two pathways (MEK and FLT3) has a profound effect on AML cell proliferation. To further evaluate this pathway, we tested the effect of treating MV4-11 cells with MEK inhibitor U0126, alone or in combination with FLT3 inhibitor. Our results showed that U0126 treatment significantly (p ≤ 0.01) reduced the viability of the MV4-11 cells in a dose-dependent manner (Fig. 6A). Consistent with previous reports, our research findings showed that U0126 inhibited the FLT3-ITD AML cell line, MV4-11. Based on these results, we further evaluated the combination effect of both MEK inhibitor and FLT3 inhibitor on MV4-11 cells in vitro. Our results showed that the combination treatment of both drugs enhanced the inhibition of cell viability (Fig. 6B). These findings have important implications for clinical treatment of leukemia because single-agent FLT3 inhibitors for patients with the FLT3-ITD mutation have been unsustainable. The mechanism of resistance to FLT3 inhibitors was associated with the aberrant activation of parallel signaling pathways such as MAPK and AKT^42,43^. Combination drug treatment of MEK-ERK1/2 and FLT3 inhibitors was effective against tyrosine kinase inhibitor-resistant AML^44,45^. Therefore, our result of MEK and FLT3 inhibitors on AML provides proof-of-concept that drug combinations can be readily tested using the zebrafish platform, enabling quick assessment of efficacy and potential toxicity to the organism can be evaluated.

## Discussion

Groups of xenotransplanted embryos can be treated with pharmaceutical drugs to identify tumor-drug sensitivities through fluorescence imaging or ex vivo quantification of cell proliferation after the dissociation of xenotransplanted embryos. This procedure enables rapid characterization of tumor-drug sensitivities within a 3–7 day time frame, which is crucial for high-throughput drug screening and rapid drug efficacy testing for personalized medicine. Such a high-throughput platform for pre-clinical drug testing may offer unique advantages when screening large numbers of drug combinations targeting different leukemia subtypes with distinctive tumor biology^31^. Based on these advantages, our goal was to validate the zebrafish xenograft model for several existing drug candidates against leukemia. Here, we evaluated the drug effect on cancer cells in the zebrafish xenografts, demonstrating that the cancer cells could adapt to the host environment and proliferate inside the zebrafish. To evaluate the transplanted K562 cells in zebrafish xenografts, we analyzed the leukemia growth by cell counting after embryo dissociation at 3 dpf and 5 dpf of the zebrafish xenografts. The results confirmed that leukemia cells proliferate significantly inside the zebrafish host. Thus, the zebrafish/leukemia xenograft model functions in a similar manner to the mouse model—but requires far less time. We have investigated the in vitro drug effects previously reported for leukemia cells using our zebrafish xenograft model. For this proof-of-concept work, we selected the well-established drugs IM and ATO for testing on K562 cells. Both drugs inhibited K562 cell proliferation in zebrafish xenografts, confirming the results established in the in vitro experiments. In the zebrafish xenografts, the dosing of IM (1 μM and 3 μM) and ATO (2 μM and 6 μM) are comparable to that of murine xenografts of IM (100–150 mg/kg or 200–300 μmol/kg)^46^ and ATO (5–10 mg/kg or 25–50 μmol/kg)^47^. The zebrafish xenografts responded to the targeted and chemotherapy drugs, which were well tolerated with no noticeable toxicity to the zebrafish. Examples of zebrafish embryo morphology (in bright field and TRITC fluorescence; see Figs. 1, 4 and 5**)** showed that both control embryos and embryos in various drug treatments were free of gross morphological abnormalities. Our data further confirmed that the zebrafish xenograft model worked similarly to other in vivo models.

Our long-term goal is to develop the zebrafish/leukemia xenograft as a robust pre-clinical model for prioritizing potential drug treatments for leukemia. To validate the zebrafish as a potential in vivo pre-clinical model, we xenotransplanted different human cells, including cancer cells and normal human primary cells (CD34^+^ HSPC and CD3^+^ T-cells; Fig. 2B,C). All human cells engrafted well and proliferated inside the zebrafish. We showed for the first time that the engrafted human HSPC and T-cells can persist at least 15 dpf (13 days after transplantation) in zebrafish embryos. In future studies, we will use this model to further investigate different developmental properties of human HSPC. These data confirmed that the zebrafish in vivo model enabled engraftment of both human leukemia cell lines and human primary cells, which will enable testing of off-target hematologic toxicity. The success of this zebrafish model may provide the groundwork for the future development of a superior pre-clinical high-throughput model. Indeed, this model can be used to efficiently evaluate potential treatment options for leukemia subtypes by transplanting the cells of interest and then studying the efficacy and safety of combo-drug regimens for that particular subtype^48–50^. Future study of successful engraftment of human HSPC into zebrafish leukemia xenografts may simultaneously evaluate potential hematotoxicity and drug efficacy in cancer therapy.

Cell proliferation can be visualized in real-time using fluorescence imaging of the zebrafish xenografts, which is an additional advantage of using zebrafish in vivo model for leukemia drug testing. To demonstrate this application, we evaluated this zebrafish model for targeted drug treatment against leukemia cells. The mitogen-activated protein kinase (MAPK) pathway is a key integration point along the signal transduction cascade that links diverse extracellular stimuli to proliferation, differentiation, and survival^38^. Inappropriate MAPK activation has a potential role in transforming myeloid cells into leukemic cells. Internal tandem duplications of the FLT3 receptor also result in constitutive MAPK activation and the autonomous growth of myeloid cell lines and primary AML samples^51^. MEK inhibitors have been shown to inhibit leukemia cell proliferation in zebrafish xenografts and cell culture. We confirmed these findings using the MEK inhibitor U0126 on two leukemia cell lines. In addition, a cytotoxic drug (Ara-C) and an epigenetic modifier drug (Aza) were used in this model. Our study confirmed these drugs reduced leukemia cell progression without obvious toxicity to the zebrafish; moreover, the treatments caused no adverse effect on heart beat or differentiation of neural and blood tissues during development. These rapid screening methods with drugs of different therapeutic mechanisms can be used to prioritize and select the best individual drug or combination of drugs for different subtypes of leukemia (e.g., based upon mutation status). We further evaluated the effect on MV4-11 AML cells of treatment with MEK inhibitor, both individually and in combination with FLT3 inhibitor gilteritinib, using a cell culture assay. The results demonstrated that treatment with combined MEK inhibitor and FLT3 inhibitor resulted in enhanced inhibition of MV4-11 cell proliferation than MEK inhibitor alone (Fig. 6A,B). Our result was consistent with a recent study demonstrating that trametinib, another MEK inhibitor, could enhance the activity of midostaurin, a FLT3 inhibitor^45^. Because both the MEK and FLT3 pathways play a role in AML cell progression, inhibiting both pathways may be a potential strategy to develop a future therapeutic regimen to combat AML.

Zebrafish have been used for high-throughput screens of chemical libraries; however, no large screen has been reported in the zebrafish/tumor xenograft model. Even fewer studies examined the zebrafish/leukemia xenograft model; further, most of these were evaluated using whole-mount imaging microscopy. Here, we performed dissociation of the xenograft embryos and used a quantitative method to evaluate the number of human cells that proliferated inside the zebrafish host. A few other studies on xenotransplantation of human HSPC have been successful in the immunocompetent *casper* zebrafish mutant; however, cells did not survive 24 h post-transplant^24,52^. Even with transgenics that express cytokines such as CXCL12, SCF, and GM-CSF in *casper* zebrafish background, these cells survived past 48 hpi and began losing cytoplasmic dye^52^. Our results demonstrate that human CD34^+^ HSPC and CD3^+^ T cells can survive inside the immuno-deficient zebrafish host (SCID ZF) for at least 15 dpf (Fig. 2B,C). Thus, the zebrafish xenograft model prosents an attractive potential alternative to murine xenograft models for analysis of human hematopoietic cells. In our study, we xenotransplanted human leukemia cells via the duct of Cuvier of SCID zebrafish embryos at 2 dpf and incubated at 33 °C, after which we routinely analyzed after 3 days at 5 dpf. Other studies used 32–35 °C incubation temperature and performed the transplantation in 2 dpf embryos^25,53,54^. We chose 33 °C because we found that our SCID zebrafish/leukemia xenografts survived better and were more healthy at this incubation temperature. Our results also showed that chemotherapeutic drugs exert anti-leukemia activity without obvious toxicity in zebrafish xenografts.

## Methods

### Chemicals and reagents

Primary Human CD34^+^ cells from Yale Cooperative Center of Excellence in Hematology (YCCEH, Yale University) were immunomagnetically isolated for CD34^+^ as described^55^ from granulocyte colony stimulating factor–mobilized peripheral blood mononuclear cells from healthy donors. Primary human T-cells were received from UNC Chapel Hill; StemSpam, Methocult Media, MesenCult MSC Basal Media, ACF, MyeloCult (H5100) media, and BIT 9500 serum substitute were purchased from stem cell technologies (Vancouver, Canada). Growth factors IL-3, IL-6, FLT3L, SCF, IL-2, IL-7, and IL-15 were all obtained from ConnStem (CT, USA). Gilteritinib (MW 552.7) was purchased from Medchemexpress (NJ, USA), azacytidine (MW 244.2) from Selleck Chemical (TX, USA), arsenic trioxide (MW 197.8) from Alfa Aesar (MA, USA), cytarabine (MW 243.2) from Acros Organics (NJ, USA), and imatinib (MW 589.7) and U0126 (MW 380.5) from MilliporeSigma (MA, USA). Vibrant CM-DiI was purchased from Invitrogen (CA, USA).

### Zebrafish husbandry and embryo incubation

SCID zebrafish in *casper* background with DNA-dependent protein kinase catalytic subunit (*prkdc*) mutant lines^1^ were kindly provided by Dr. David Langenau (The Massachusetts General Hospital, Boston, MA). Zebrafish embryos were staged and maintained following standard protocols^56,57^ and in accordance with North Carolina Central University IACUC guidelines. Zebrafish embryos were maintained as described^6^. Briefly, they were incubated in 0.3X Danieau’s solution at 28.5 °C; phenylthiourea (PTU, 30 µg/ml) was added around 8 hpf (75% epiboly stage) to inhibit pigment formation, specifically to inhibit the black pigment of the retina in *casper* background.

### Cell culture

All human leukemia cell lines MV4-11, K562 (ATCC, Manassas, VA), and MOLM-13 (AddexBio, San Diego, CA) cells were purchased and authenticated based on short tandem repeat profiling/analysis (Cell Line Authentication Analysis, DNA Analysis Facility, Duke University). K562 and MOLM-13 were cultured in RPMI 1640 (Gibco, Thermo Fisher Scientific, USA) and 1% penicillin/streptomycin (Gibco, Thermo Fisher Scientific, USA), K562 with 10% FBS (Gimini bio-product, USA) and MOLM-13 with 20% FBS. MV4-11 cells were cultured in IMDM (Gibco, Thermo Fisher Scientific, USA) and 1% penicillin/streptomycin with 10% FBS. MV4-11 cells usually grew slightly more slowly than K562 and MOLM-13. Human CD34^+^ cells were expanded in serum-free medium (Stemspan, StemCell Technologies) containing growth factors IL-3 (20 ng/mL), IL-6 (20 ng/mL), FLT3 ligand (100 ng/mL), SCF (100 ng/mL), and 10% BIT. Human T-cells were cultured in RPMI 1640, 10% FBS, 1% penicillin/streptomycin with growth factor IL-7 (10 ng/mL) and IL-15 (10 ng/mL).

### In vitro cell viability assays

1 × 10^4^ cells/well were seeded with complete media in 96-well plates. After 24 h the cells were treated with varying concentrations of different drugs (U0126 and Gilteritinib). After 72 h treatment, 100 µL of total volume of the media along with the cells were collected in another black-walled 96 well plate. 100 µL of the 1X PBS along with live and dead assay dye (EarlyTox live and dead assay kit from Molecular Devices) according to the prescribed protocol. The live and dead stained cells were analyzed by using the Image Xpress Pico Automated Cell Imaging System (Molecular Devices). The data were tabulated and plotted for percentage of viable cells.

### Zebrafish/leukemia xenografts and drug treatment

The exponentially growing cells were stained with Vibrant CM-DiI dye one day before injection into zebrafish embryo. On the day of injection, cells were counted and resuspended at a concentration of 1 million cells per 10 µL of RPMI media with 1% FBS. The suspended cells were injected via the duct of Cuvier into the circulation of 2 dpf zebrafish embryo using a FemtoJet microinjector (Eppendorf) with constant injection pressure and injection time. The injection volume and cell suspension was calibrated to deliver 400–500 cells/injection in each embryo. A micromanipulator MM3301-R, (World Precision Instrument Company, Novato, CA, USA) was used or microinjections using borosilicate glass capillaries (1.0 mm in diameter), which were pulled to inner and outer diameters of 18 and 20 μm, respectively, using a Micropipette Puller (Model P-97 from Sutter Instrument Company, Novato, CA, USA). After transplantation, the embryos were immediately placed at 33 °C. After an hour, embryos with transplanted human cells in the blood circulation were screened and embryos with abnormal morphology or inadequate transplantation with only few fluorescent human cells in the embryos (5% to 10% of grafted embryos) were removed and euthanized. After sorting, embryos were randomly chosen for each group, incubated at 33 °C, and systemically exposed to different concentrations of dissolved pharmacological compounds in the embryo medium for three days. The concentrations of compounds were selected based on pilot experiments showing that they do not cause any developmental defects or toxicity to zebrafish embryos. These concentrations were comparable to mouse studies^58–62^. Control groups were treated with DMSO at the same end concentration. The SCID zebrafish have a mutation in the *prkdc* gene, which is required during lymphocyte development for immunoglobulin and T-cell receptor gene assembly. Zebrafish T-cells appear as early as 3 dpf embryo that thymic rudiment is fully developed and colonized by T lymphocyte progenitors^63–67^. Although the zebrafish immune system appeared not fully developed at this early stage, using SCID zebrafish embryos can eliminate any potential effect of developing T-cell mediated host vs. graft immune response. Zebrafish were euthanized using overdosed tricaine (3-amino benzoic acidethylester) according to approved animal protocol. All experimental procedures using zebrafish xenograft research were approved by the Institutional Animal Care and Use Committee of the North Carolina Central University (NCCU IACUC Protocol No. TCL-07-14-2008, Leung), and all experiments were performed in accordance with relevant guidelines and regulations, in compliance with the ARRIVE guidelines (http://www.nc3rs.org.uk/page.asp?id=1357).

### Dissociation of xenografted zebrafish for cell analysis

The control group and drug-treated zebrafish embryos were collected separately and placed into 1.5 ml tubes (2, 3, 4, or 10 embryos/tube for quantitation experiments). The embryos were dissociated using collagenase (3 mg/ml in PBS), incubated in 37 °C for 30 min, and agitated by pipetting 15 times. This procedure was repeated for 2 additional cycles. After dissociation, the process was stopped by adding 25% FBS in PBS; cells were then collected by centrifugation. Dissociated cells were suspended in 10% FBS in PBS, transferred into black walled 96-well plates, and then subjected to cell counting using ImageXpress Pico (Molecular Devices). Plates were centrifuged at 500 rpm (50×*g*) for 2 min to bring suspended cells to the bottom. The "Cell Counting" application of ImageXpress with bright field and TRITC fluorescent setting were selected. Once inside the ImageXpress chamber, plates were subjected to cell counting of red fluorescently labeled human cells within the selected range of cell sizes according to manufacturer instructions. The counted cells were analyzed and a graph was plotted for total number of stained cells in the drug treatment vs. control groups. Undissociated embryos from each group were documented for representative images by using the ImageXpress Pico.

### Statistical analysis

Statistical analysis was performed using one-way ANOVA to determine statistical significance among the groups, followed by Student’s t-test to determine p values. Data were expressed as the average ± standard error of the mean of biological replicates for each experiment. The results were confirmed by repeats of two independent experiments. The observed differences were regarded as significant if the calculated two-tailed probability (*p*) values *p ≤ 0.05, **p ≤ 0.01 and ***p ≤ 0.001.

### Ethical approval

All experimental protocols and procedures are approved by and conformed to the guidelines of the Animal Care and Use Committee of North Carolina Central University (Durham, NC). (NCCU IACUC Protocol # TCL-07-14-2008).

## Data Availability

All data generated or analyzed during this study are included in this published article and supplementary figures.
